# Curcumin-Loaded Nanomaterials as Carriers for Photodynamic Therapy Against Cancers

**DOI:** 10.3390/gels11100833

**Published:** 2025-10-17

**Authors:** Chuanshan Xu, Siu Kan Law, Albert Wing Nang Leung, Mei Feng

**Affiliations:** 1 Guangzhou Municipal and Guangdong Provincial Key Laboratory of Molecular Target & Clinical Pharmacology, The NMPA and State Key Laboratory of Respiratory Disease, School of Pharmaceutical Sciences, Guangzhou Medical University, Guangzhou 511436, China; xcshan@163.com; 2 Independent Researcher, Hong Kong SAR, China; siukanlaw@hotmail.com; 3 School of Graduate Studies, Lingnan University, Tuen Mun, Hong Kong SAR, China; albertleung@ln.edu.hk

**Keywords:** nanogel, curcumin, photodynamic therapy, cancer

## Abstract

Cancer is a prevalent disease worldwide. Photodynamic therapy (PDT) is a non-invasive and highly targeted treatment for certain types of cancer. Recently, it has been combined with nanotechnology, e.g., nanogel, for enhancing its therapeutic efficacy. “Curcumin” is a more typical naturally occurring photosensitizer (PS) in PDT, due to its wide range of pharmacological activities, including anticancer, anti-inflammatory, antibacterial, and antiviral effects. However, curcumin has low bioavailability, limiting its therapeutic potential, which can be improved with the help of nanotechnology. Growing evidence has shown that curcumin-loaded nanogels have their specific functions, such as drug delivery and cancer targeting. Curcumin-loaded nanogel has overcome the limitations of free curcumin, such as solubility and controlled release, with the help of these, as they provide a multifunctional platform to enhance the therapeutic effects of PDT. However, it is still required to further investigate the combinations of curcumin, nanogel, and PDT. Much more work needs to be done, including safety assessments of curcumin-loaded nanogel with PDT delivery systems, long-term toxicity on the human body, and optimization of dosage for curcumin, nanogels, PS, light parameters, and delivery mechanisms for the PDT process, as well as the exploration of tumor-specific targeting and immune response for humans, for example, adverse drug reactions or drug to herbal interaction.

## 1. Introduction

Nowadays, nanotechnology is a famous tool for cancer diagnosis and detection [1], especially for the applications of nanoparticles, which may detect cancer cells and target them to deliver a drug for the treatment. Nanoparticles (NPs) encompass a diverse array of types, including polymeric forms such as nanogels, nanofibers [2], and liposomes [3]; metallic variants like gold nanoparticles (GNPs) [4], silver nanoparticles (AgNPs) [5], and calcium nanoparticles (CaNPs) [6]; as well as carbon-based structures including carbon nanotubes (CNTs) [7], graphene [8], and semiconductor-based quantum dots (QDs) [9].

They are employed for their exceptional sensitivity, precision, and multifunctional versatility. Notably, certain NPs—such as nanobubbles and oxygen-releasing polymers—play a critical role in alleviating hypoxia within the tumor microenvironment. Their intrinsic theranostic properties also make them valuable in photodynamic therapy (PDT), where they enhance therapeutic efficacy through simultaneous diagnostic and treatment capabilities [10].

This review article explores the synergistic application of nanogels and photodynamic therapy (PDT) in cancer treatment, with a particular emphasis on the integration of Chinese Herbal Medicine (CHM). It describes the role of curcumin—a bioactive compound derived from turmeric—as a natural photosensitizer (PS) within nanogel-based delivery systems. Distinct from existing literature, this concentrates on curcumin-loaded nanogels as targeted carriers for PDT, aiming to enhance therapeutic efficacy, bioavailability, and tumor selectivity, as well as examines the physicochemical properties of nanogels, their responsiveness to tumor microenvironments, and the potential of CHM-derived PSs to overcome limitations associated with conventional PDT agents.

### 1.1. Nanogels

Nanogels are a kind of soft, flexible gel-like nanomaterial with sizes ranging from 20 to 200 nanometers (nm). Similar to other nanomaterials, it has lots of precision tasks, including drug delivery, diagnostics, and tissue engineering. PDT is a less invasive treatment usually applied for cancer therapy. This becomes more accurate with the help of nanogels. CHM has acted as a PS in PDT for nearly two decades. However, there are still some problems that require further development and investigation.

Nanogels have provided a hydrophilic platform for the encapsulation of molecules, which enables the nanogels to respond selectively when subjected to external stimuli, and are applied to a wide range of biological and therapeutic applications. The characteristics and properties can maintain biocompatibility, biodegradability, and protect sensitive cargo like proteins or nucleic acids from degradation. They are usually soft materials that form a cross-linked network with small molecules, bio-macromolecules, and inorganic nanoparticles. The soft materials are encapsulated through a semipermeable and selective polymer. A polymer acts as a carrier that releases the corresponding components to a desirable site for therapeutic actions under an external stimulus environment, such as light exposure, temperature, pH changes, enzyme activity, and redox conditions [11].

### 1.2. Photodynamic Therapy (PDT)

PDT has three major components, including the light source, PS, and ROS. Light source parameters, such as light source, power density, wavelength, frequency, and photosensitizer types, concentration, dosage, temperature, and incubation time, influence the effectiveness of PDT [12].

“Light exposure” is a critical criterion for PDT, which has lasers, LEDs, broadband lamps, and daylight, generally in the red region or above the visible spectrum (≥600 nm). Light selectively activates the PS to generate reactive oxygen species (ROS), which accumulate in malignant tissues, causing photocytotoxicity to neoplastic cells and tumor regression. It also involves the processes of apoptosis, necrosis, autophagy, vascular shutdown, and immune activation. Apoptosis and necrosis damage the mitochondria, lysosomes, and cell membrane; autophagy causes the self-digestion of cellular organelles by using an enzyme, “autophagosomes”; vascular shutdown breaks down the nutrient supply to cause damage to the blood vessels; and the release of tumor antigens stimulates an immune response [13].

“PS” is designed to selectively accumulate in abnormal cells, such as cancerous cells, sparing healthy ones. It acts as a target agent and transfers energy to surrounding oxygen molecules when excited by the light source of a specific wavelength.

The production of “ROS” has two types of mechanisms. Type I is the electron transition from the PS at the ground state, excited to the singlet excited state, and then to the triplet excited state. The free radicals, such as hydroxyl radical HO·, and hydroperoxyl radical HOO·, are formed by the excited PS molecules interacting with the substrate during electron transfer [14]. Conversely, Type II is the excited PS molecule that transfers energy to molecular oxygen to produce highly active singlet oxygen [15]. ROS are the cytotoxic agents that lead to cellular destruction in PDT. These Type I and II mechanisms can produce ROS in nanogel-based PDT. The Type I mechanism is much favored and suitable for the treatment of hypoxic tumors. The effectiveness of PDT depends on its nanogel strategies, including the type of PS to generate the percentage yield of ROS, synergistic effects on dual modes in Type I and II mechanisms, and pH-responsive properties that offer an acidic tumor microenvironment [16].

### 1.3. Photosensitizers Isolated from Chinese Herbal Medicine (CHM)

The CHM compounds are derived from plants. It is natural and tends to be non-toxic, biodegradable, and well-tolerated within the human body compared to synthetic PS agents [17]. Some CHM PS molecules contain chromophores such as aloe emodin [18,19,20], berberine [21,22,23], and hypericin [24,25,26], which generate ROS under photoactivation to fight undesirable cancers. This article focuses on the most famous CHM, “curcumin,” and may discuss it in detail.

## 2. Methodology

WanFang Data, PubMed, Science Direct, Scopus, Web of Science, Springer Link, SciFinder, and China National Knowledge Infrastructure (CNKI) are the nine electronic databases used and searched for this review within the last 20 years without language limitations. The relevant papers, which consist of nanogel, curcumin, and photodynamic therapy, are reviewed, analyzed, and summarized.

## 3. Past Research Progress

Some representative examples of nanotechnology for cancer diagnosis and detection, as well as photosensitizers isolated from CHM against cancer, are listed in Table 1 and Table 2, respectively.

## 4. Curcumin

**Figure 1:**
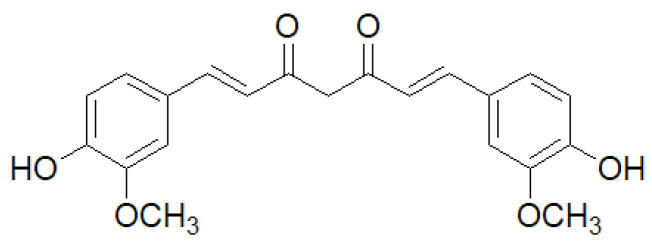
Chemical structure of curcumin.

This yellow polyphenolic compound was extracted from the rhizome of *Curcuma longa* L. (turmeric), and it has been utilized as a food coloring and culinary ingredient as well as a component of Chinese medicine (Figure 1) [27].

### 4.1. Curcumin for PDT

**Figure 2:**
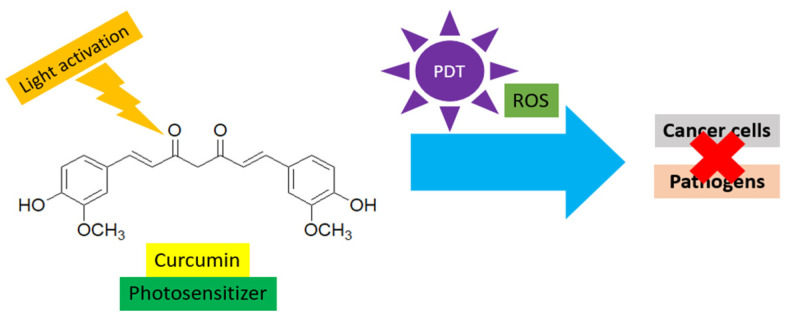
Curcumin acts as a natural PS in PDT against cancer cells and pathogens.

Curcumin acts as a natural PS in PDT. It absorbs light between 300 and 500 nm, making it suitable for activation by visible light sources to produce singlet oxygen and other ROS, which are lethal to cancer cells and pathogens (Figure 2) [28].

More importantly, curcumin is non-toxic and highly biocompatible. It possesses a wide range of pharmacological activities, and its general applications in medical practice include (a) anti-inflammatory, (b) anti-oxidation, (c) lipid regulation, (d) antiviral, and (e) anticancer applications with hypotoxicity and minor adverse reactions [29]:

(a) binds to TLRs and regulates downstream NF-κB, MAPK, AP-1, and other signaling pathways, thereby regulating inflammatory mediators and treating inflammatory diseases [30].
(b) maintains the redox balance through the Keap1-Nrf2/ARE, NF-κB, NOX, and MAPK signaling pathways, which are involved in scavenging ROS, enhancing the activity of antioxidant enzymes, inhibiting lipid peroxidation, and chelating metal ions [31].
(c) mitigates lipid metabolism disorders by lowering ROS accumulation, that is, reducing fat storage, enhancing fatty acid absorption, and boosting insulin sensitivity through modulation of the oxidative stress pathway [32].
(d) serves as a veridical agent via attacking and disrupting the integrity of viral membrane envelopes that influences viral replication machinery in two ways, including direct interference with the viral replication machinery and modulation of host cell signaling pathways, such as NF-κB, PI3K-AKT, and Jab-1 [33].

However, this review focuses on the anticancer effect of curcumin, which has been widely studied and utilized as a PS of PDT for various cancer therapies (Table 3). However, curcumin exhibits poor oral bioavailability and undergoes rapid metabolism, along with other pharmacokinetic limitations that hinder its therapeutic efficacy. PDT activates curcumin to induce targeted oxidative stress in cancer cells, leading to arrest of the cell cycle growth and ultimately apoptosis [34]. The possible apoptotic pathways for anti-cancer effects of curcumin on cell signal transduction include PI3K, Akt, mTOR, ERK5, AP-1, TGF-β, Wnt, β-catenin, Shh, PAK1, Rac1, STAT3, PPARγ, EBPα, NLRP3 inflammasome, p38MAPK, Nrf2, Notch-1, AMPK, TLR-4, and MyD-88 [35].

### 4.2. Nanotechnology of Curcumin for PDT

Nanotechnology has been widely used in PDT. It has great advantages in drug delivery, diagnosis, and therapy of illness. These act as carriers or active agents, enhancing the stability and solubility of PSs, improving their targeting efficiency, and facilitating the co-delivery of anticancer effects [45]. Most PS (e.g., curcumin) is hydrophobic, resulting in low solubility in water and reducing the efficiency of ROS production upon light irradiation [46]. Nanotechnology focuses on developing drug delivery systems, such as polymeric nanocarriers, nanoemulsions, mesoporous silica nanoparticles, liposomes, magnetic nanoparticles, chitosan/tripolyphosphate (TPP) nanoparticles, and nanocomposites for PSs to further improve the effectiveness of PDT. A list of representative examples for nanotechnology of curcumin with PDT against cancer is shown in Table 4.

## 5. Nanogel for PDT

PDT has gained considerable interest because of its benefit of localized treatment. Nonetheless, the indiscriminate distribution of photosensitizers, including solubility and bioavailability, limits their broader application in cancer therapy. Nanogels have been developed to reduce these shortcomings and enhance the effectiveness of PDT through the selective drug delivery system (Table 5).

### 5.1. Nanogel with Curcumin

As discussed above, curcumin has some disadvantages in clinical applications, but these may be overcome with the help of a nanogel through the development of a delivery system to enable controlled and sustained release. Nanogels also increase the bioavailability, such as solubility and stability. More importantly, nanogels have the protective function to encapsulate curcumin for preventing degradation (Table 6).

### 5.2. Curcumin-Loaded Nanogel for PDT

**Figure 3:**
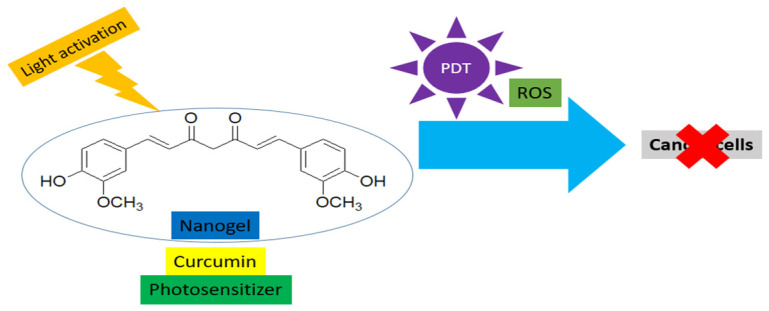
Nanogel-encapsulated curcumin with PDT against cancer cells.

Nanogels overcome the limitations of curcumin and provide a platform for PDT. This controls the targeted release behavior and tumor specificity for the generation of ROS to kill cancer cells during the PDT process (Figure 3). It may involve a dual mechanism of action, such as inhibiting survival pathways like NF-κB and TNF-α. Two representative examples of the curcumin-loaded nanogel for PDT are presented in Table 7.

Despite their promising potential, curcumin-loaded nanogels face several challenges in PDT. One major issue is the low water solubility of curcumin, as its hydrophobic nature limits effective dispersion in biological fluids even when encapsulated within nanogels. Meanwhile, curcumin is highly sensitive to pH, leading to rapid degradation under physiological conditions. Therefore, minimizing degradation is critical for maintaining therapeutic efficacy. Nanogels are generally considered biocompatible, and their long-term toxicity, especially in combination with curcumin, remains insufficiently understood and warrants further investigation.

## 6. Discussion

Based on the above review, several questions must be considered, which are about curcumin nanogels with PDT against cancer:

(1) Why use curcumin as a PS in PDT?
(2) What are the purposes of using nanotechnology and nanogels?
(3) Would nanotechnology and nanogels help to improve the bioavailability of curcumin and the effectiveness of PDT?
(4) Are there any effective relationships for nanogel, curcumin, and PDT?
(5) What are the similarities between upconversion nanoparticles (UCNPs) and nanogels?
(6) Why combine PDT with Phototherapy Therapy (PTT)?
(7) What are the future aspects for the nanogel of curcumin with PDT?

These doubts can be discussed as follows in a point-by-point format.

Firstly, curcumin is a famous traditional Chinese herbal medicine used as a PS in PDT for a long time. Basically, it generates ROS, including singlet oxygen and hydroxyl radicals, when exposed to blue light in the wavelength range from 300 to 500 nm, which may disturb or destroy the cancer cells. However, the bioavailability of curcumin is not good as well, with lower solubility and stability, etc., affecting the effectiveness of PDT. Thus, it is usually combined with the help of nanotechnology or nanogels to overcome these limitations.

Nanotechnology can enhance the properties of PS (e.g., curcumin), including poor water solubility, low bioavailability, limited tumor selectivity, photobleaching, and instability. Nanogels are a sub-branch of nanotechnology, which have the same functions, and have several specific features, such as biocompatibility, stimuli responsiveness, controlled release, protection of payload, and excellent hydrophilic or hydrophobic loading capacity. They are specifically designed as soft, water-swollen polymeric nanoparticles. The PS (e.g., curcumin) is usually encapsulated through the polymer.

When curcumin is combined with nanogels during PDT, it addresses its limitations and amplifies the therapeutic potential. The main advantages of nanogels are reduced photobleaching, high biocompatibility, and low toxicity. Since curcumin is photosensitive, it can be protected from degradation by being encapsulated in nanogels. The backbone of a nanogel is a polymer, such as chitosan, chitin, dextran, and gelatin, which are highly biocompatible and non-toxic. These help to encapsulate the curcumin in a nanogel delivery system to improve the effect of therapeutic efficacy on PDT. One of the major disadvantages is tumor hypoxia, which can reduce PDT efficacy, even if nanogels deliver the photosensitizer efficiently. The effectiveness of the therapeutic potential of PDT can be enhanced by optimizing light, tissue oxygenation, and the inherent properties of the photosensitizers, as well as the selectivity, bioengineering, and subcellular or organelle targeting [79]. Its strategies include the modification of using nanoparticles to deliver drugs and light, employing light sources with better tissue penetration, or generating light within the tissue [80]. It is more effective to use near-infrared (NIR) light in PDT to penetrate deeper into tissues than visible (blue) light for internal treatment. Curcumin has a short wavelength with the help of UCNPs, which convert NIR light into visible wavelengths to activate traditional photosensitizers inside tissues or organs [81].

There is some evidence of using UCNPs. Lakshmanan et al. described the development of a curcumin nanoscale formulation, a promising anticancer herbal compound, to enhance its cellular and systemic delivery in in vitro and in vivo models. This UCNP-PLGA-nanocurcumin exhibited emission at 480 to 800 nm and facilitated organ-specific biodistribution, with predominant accumulation in the liver and lungs, supporting its application in imaging-based diagnostics [82]. Jing et al. developed UCNPs-F127@Cur, which significantly induced apoptosis, elevated intracellular ROS production, suppressed the expression of pluripotency-associated genes in glioma stem cells, and effectively inhibited their in vivo tumorigenic growth following transplantation under 980 nm excitation [83]. Chen et al. also identified MnO_2_-decorated upconversion nanoparticles with curcumin (UCSMn-Cur) that degraded into manganese ions (Mn^2+^), referring to the reaction with H_2_O_2_ in the acidic tumor microenvironment, producing oxygen and facilitating curcumin release under the transformation of infrared light to visible light of 450 and 475.5 nm to induce tumor cell apoptosis [84]. Ye et al. indicated that upconversion nanoparticles conjugated with curcumin (UCNPs-curcumin) might have the potential to convert near-infrared (NIR) light into wavelengths suitable for curcumin excitation. These UCNPs-curcumin were activated in the lung under NIR irradiation and strengthened their antibacterial effects on methicillin-resistant *Staphylococcus aureus* [85]. These findings underscore the potential of UCNP-nanogel-curcumin platforms for deep-tissue PDT, combining therapeutic and diagnostic capabilities in a single system.

Certain properties of curcumin can be further enhanced via nanogel encapsulation, owing to the advantages of multifunctionality and photodynamic synergy compared to UCNP-based systems. Nanogels have multiple functions: delivery, controlled release behavior, and protection of curcumin in one single system. It also increases curcumin’s therapeutic action directly, and does not only focus on activation, which is a derived benefit. When nanogels are combined with PDT, it can further enhance its functions, especially in terms of tumor targeting and selectivity to ensure that PS is activated only in diseased tissues or cancer cells, sparing healthy tissues or cells.

Over the past decades, PDT has been extensively employed and often combined with photothermal therapy (PTT) to enhance therapeutic outcomes by overcoming the limitations of each modality and improving safety and efficacy [86]. Different from PDT, PTT converts light energy into heat energy for the treatment of cancer through the temperature increases without bleeding and fast recovery [87]. This approach typically employs nanoparticles embedded within tumors as exogenous energy absorbers, converting laser light into localized heat to ablate cancer cells, thereby inducing necrosis and triggering pro-inflammatory responses [88].

PDT is less effective under oxygen-deficient conditions, whereas PTT can augment oxygen supply and boost therapeutic outcomes. Urazaliyeva et al. developed a novel nanomedicine by integrating an organic photothermal agent with photosensitizer, forming a colocalized nanoplatform to enhance phototherapeutic efficacy in cancer treatment, which demonstrated superior cytotoxicity and apoptosis induction under hypoxic conditions, outperforming nanoparticles designed for PTT alone [89], since the NPs might absorb a longer wavelength at 635 nm from NIR to activate the photothermal agent for producing heat energy to induce apoptosis. Zang et al. reported a DNA nanogel-coated polydopamine nanoparticle exhibiting high drug loading for combined chemo-photothermal cancer treatment. The nanogel or hybrid processed a high drug loading and photothermal conversion efficiency for the tumor sites [90]. Xiao et al. also developed a hybrid hyaluronic-acid-based nanogel system incorporated with polypyrrole and doxorubicin to achieve controlled and optimized DOX release within the targeted tumor microenvironment [91]. Chang et al. designed a low-toxicity, biodegradable photothermal agent via copolymerization of sodium copper chlorophyllin with a nanogel of N-[3-(dimethylamino)propyl]methacrylamide. This was a potential photothermal cancer therapy under a green laser with a 532 nm wavelength [92].

Growing evidence has shown that PDT and PTT complement each other, enhancing their therapeutic effects. These systems enhance drug delivery by modulating tumor perfusion, vascular and extracellular matrix permeability, and the overall tumor microenvironment. Additionally, their non-invasive nature reduces the risk of off-target damage compared to systemic therapies. More importantly, both are non-ionizing radiation with a lower chance of developing secondary cancer [93].

Guo S et al. reported a graphene oxide (GO) platform dual-functionalized with folic acid (FA) and chlorin e6 (Ce6) for targeting PTT/PDT, enabling rapid penetration into cancer cells and macrophages and resulting in enhanced cytotoxicity across diverse cell types implicated in cancer and related diseases [94].

Kong et al. designed ICG@SANPs-cRGD nanoparticles, integrating indocyanine green and cyclic RGD peptide for synergistic PTT/PDT against breast cancer, demonstrating potent inhibition of tumor cell growth and motility, along with apoptosis- and necrosis-mediated cytotoxicity [95].

Therefore, the development of nanogels in the future will be more inclined to apply PDT and PTT, as they can enhance their functional systems for therapeutic effects, making them more accurate and reliable.

## 7. Conclusions

A curcumin-loaded nanogel with PDT is effective for the treatment of cancer from Type I and II mechanisms through different signaling pathways, including PI3K, Akt, mTOR, ERK5, AP-1, TGF-β, Wnt, β-catenin, Shh, PAK1, Rac1, STAT3, PPARγ, EBPα, NLRP3 inflammasome, p38MAPK, Nrf2, Notch-1, AMPK, TLR-4, and MyD-88. The nanogel operates as a carrier or active agent, improving the stability and solubility of curcumin and targeting capabilities, and also co-delivering additional anticancer medications. However, extensive research is still needed, especially to assess the safety of curcumin-loaded nanogels used in PDT. It should determine the long-term toxicity within the human body, its tumor-specific targeting, and immune response. Clinical studies are the next milestone, during which the therapeutic effects on the delivery system for PDT would be assessed.

## Figures and Tables

**Table 1:** Nanotechnology for cancer diagnosis and detection.

	Study	Type of Nanoparticle (NP)	Type of Cancer	Consequence	Reference
1	Anti-epidermal growth-factor-receptor-conjugated mesoporous zinc oxide nanofibers for breast cancer diagnostics	Mesoporous zinc oxide nanofibers	Breast cancer	Mesoporous zinc oxide nanofibers, as a label-free detection of breast cancer biomarker, showed high sensitivity, and an association constant indicating a higher affinity towards the ErbB2 antigen.	[2]
2	Hyaluronic acid derivative-coated nanohybrid liposomes for cancer imaging and drug delivery	Nanohybrid liposomes	Breast cancer	Nanohybrid liposomes coated with amphiphilic hyaluronic acid-ceramide exhibited controlled release behavior, demonstrating increased therapeutic efficacy and extended drug circulation in the bloodstream.	[3]
3	Utilization of gold nanoparticles for the detection of squamous cell carcinoma of the tongue based on laser-induced fluorescence and diffuse reflectance characteristics: an in vitro study	Gold nanoparticles	Squamous carcinoma	Gold nanoparticles mixed with cancer cells produced a higher fluorescence, and the optical diffuse reflectance analysis reveals that the addition of AuNPs enhances cancer detection, especially in terms of sensitivity, specificity, and accuracy.	[4]
4	Exploring the anticancer potential of green silver nanoparticle–paclitaxel nanocarrier on MCF-7 breast cancer cells: an in vitro approach	Silver nanoparticles	Breast cancer	The nano platform synergized paclitaxel therapeutic effects with silver nanoparticle-mediated targeting, offering a promising strategy to reduce side effects and improve tumor-specific cytotoxicity, which confirmed that apoptosis induction enhanced anticancer activity.	[5]
5	Chitosan-functionalized fluorescent calcium carbonate nanoparticle loaded with methotrexate: future theranostics for triple-negative breast cancer	Calcium nanoparticles	Breast cancer	The Bax/Bcl-2 signaling pathway was activated by fluorescent calcium carbonate nanoclusters encapsulated with methotrexate and surface functionalized with chitosan, resulting in a G1 phase cell cycle arrest and subsequent late-phase death.	[6]
6	Ultrasensitive detection of MCF-7 cells with a carbon nanotube-based optoelectronic-pulse sensor framework	Carbon nanotubes	Breast cancer	The atom molecular dynamics simulations revealed that interactions between the embedded carbon nanotube and cancer cell membranes result in a less rigid lipid bilayer structure, which can facilitate carbon nanotube translocation, and the unique optoelectrical properties of carbon nanotube for unlocking the detection of a small population of cancer cells.	[7]
7	Surface-tailored graphene nanosheets targeting PI3K/Akt signaling of breast cancer cells	Graphene	Breast cancer	Graphite integrated with cetyltrimethylammonium chloride-assisted graphene nanosheets promotes PI3K/Akt-mediated apoptosis in breast cancer cells, driven by low concentrations and pronounced electrostatic repulsion.	[8]
8	Ultrasound-assisted encapsulating folic acid-based carbon quantum dots within breast-cancer-cell-derived exosomes as a co-receptor-mediated anticancer nanocarrier for enhanced breast cancer therapy	Quantum dots	Breast cancer	Methotrexate, within carbon quantum dots synthesized from folic acid, significantly improved the delivery efficacy of methotrexate directly to the cancerous cells through the binding to folate and heparan sulfate proteoglycan receptors, and increased disruption of the mitochondrial membrane potential, and consequently initiated apoptosis, culminating in the elimination of cancerous cells.	[9]

**Table 2:** Photosensitizers isolated from CHM against cancer.

	Study	Types of Cancer	CHM (PS)	Light Irradiation	Consequences	Reference
1	Inhibitory effect of aloe-emodin-mediated photodynamic therapy on human oral mucosa carcinoma in vitro and in vivo	Oral mucosa carcinoma	Aloe emodin	Blue, 430 nm	AE-PDT upregulated the levels of caspase-3 and Bax protein, but downregulated the level of Bcl-2 protein. It indicated that the AE-PDT prolonged the survival time of the tumor mouse without side effects.	[18]
2	Exploring a novel target treatment on breast cancer: aloe-emodin-mediated photodynamic therapy induced cell apoptosis and inhibited cell metastasis	Breast cancer	Aloe emodin	Blue, 430 nm	AE-PDT significantly inhibited the adhesion, migration, and invasion of MCF-7 cells, as evidenced by decreased expression of MMP2, MMP9, VEGF, and Nrf2, which is related to oxidative stress induced by the generation of ROS.	[19]
3	The effect of aloe-emodin-induced photodynamic activity on the apoptosis of human gastric cancer cells: a pilot study	Gastric cancer	Aloe emodin	Blue, 430 nm	AE-PDT exhibited an inhibitory effect on human gastric cancer cells, inducing cell apoptosis by upregulating the levels of caspase-9 and caspase-3, indicating that AE-PDT involved the mitochondrial pathway.	[20]
4	Photodynamic-therapy-triggered nuclear translocation of berberine from mitochondria leads to liver cancer cell death	Liver cancer	Berberine	Blue, 488 nm	Berberine-PDT induced cell cycle arrest and endoplasmic reticulum stress, triggering the activation of pro-apoptotic proteins on the DNA-damage-inducible transcript 3 gene, leading to inhibition of liver cancer.	[21]
5	Berberine-associated photodynamic therapy promotes autophagy and apoptosis via ROS generation in renal carcinoma cells	Renal carcinoma	Berberine	Blue, 488 nm	Berberine-PDT triggered changes in cell proliferation, tumorigenesis, and angiogenesis, as well as regulated the expression of vascular endothelial growth factor-D and human telomerase reverse transcriptase genes, and increased the generation of ROS levels, inhibiting the growth of renal carcinoma.	[22]
6	Effect of berberine associated with photodynamic therapy in cell lines	Cervical cancer	Berberine	Blue, 488 nm	Berberine-PDT increased the generation of ROS and caspase-3 activity, indicating a preferential cell death mechanism through caspase-dependent apoptosis in cervical cancer.	[23]
7	Hypericin-mediated photodynamic therapy for head and neck cancers	Head and neck cancers	Hypericin	Red, 660 nm	HY-PDT consistently demonstrated significant cytotoxicity against squamous cell carcinoma cells by the generation of ROS through apoptotic and necrotic pathways, which may enhance antitumor immunity and reduce metastasis by increasing the levels of IL-20 and sIL-6R.	[24]
8	Hypericin-based photodynamic therapy displays higher selectivity and phototoxicity towards melanoma and squamous cell cancer compared to normal keratinocytes in vitro	Skin cancer	Hypericin	Orange, 620 nm	HY-PDT was the highest phototoxic reaction, with selective uptake by cancer cells and observed proapoptotic properties compared to normal keratinocytes after irradiation.	[25]
9	Hypericin-mediated photodynamic therapy inhibits growth of colorectal cancer cells via inducing S phase cell cycle arrest and apoptosis	Colorectal cancer	Hypericin	Red, 660 nm	HYP-PDT demonstrated a significant elevation of increased Bax expression, decreased Bcl-2 expression, and upregulated the expression of cleaved caspase-9, cleaved caspase-3, as well as cleaved poly (ADP-ribose) polymerase, which induced S phase cell cycle arrest through the CDC25A/CDK2/Cyclin A pathway and apoptosis in colorectal cancer.	[26]

**Table 3:** A list of representative examples for curcumin with PDT against cancer.

	Study	Types of Cancer	CHM (PS)	Light Irradiation	Consequences	Reference
1	Assessing the effects of curcumin and 450 nm photodynamic therapy on oxidative metabolism and cell cycle in head and neck squamous cell carcinoma: an in vitro study	Head and neck cancers	Curcumin	Blue, 450 nm	Cur-PDT caused an oxidative phosphorylation metabolism impairment to cause a dissociation between cellular respiration and energy production, which results in elevated oxidative damage, diminished cell growth and viability, as well as arresting the G1 phase cell cycle—collectively suppressing the progression of head and neck cancers.	[36]
2	Evaluation of curcumin-mediated photodynamic therapy on the reverse of multidrug resistance in tumor cells	Breast cancer	Curcumin	Blue, 450 nm	Cur altered the mitochondrial membrane potential and enhanced the release of mitochondrial cytochrome c, concurrently downregulating the expression of caspase-3, -7, -9, poly (ADP-ribose) polymerase (PARP), and p-glycoprotein, which induced apoptosis in resistant MCF-7/ADM cells.	[37]
3	Effects of curcumin-mediated photodynamic therapy on autophagy and epithelial–mesenchymal transition of lung cancer cells	Lung cancer	Curcumin	Blue, 450 nm	Cur-PDT inhibited epithelial–mesenchymal transition, migration, and invasion and induced autophagy in lung cancer cells through modulation of LC3-I to LC3-II conversion and the expression levels of p62 and Beclin-1.	[38]
4	Effects of notch signaling pathway in cervical cancer by curcumin-mediated photodynamic therapy and its possible mechanisms in vitro and in vivo	Cervical cancer	Curcumin	Blue, 450 nm	Cur-PDT inhibited the proliferation and induced apoptosis of cervical cancer cells, which was linked to the suppressed expression of Notch1 and NF-κB.	[39]
5	Combination treatment with photodynamic therapy and curcumin induces mitochondria-dependent apoptosis in AMC-HN3 cells	Head and neck cancers	Curcumin	Blue, 450 nm	Cur-PDT enhanced cytotoxic and apoptotic effects on AMC-HN3 cells via a mitochondria-dependent apoptosis pathway by suppressing the generation of ROS and upregulation of caspase-3 and poly (ADP-ribose) polymerase.	[40]
6	Photodynamic therapy potentiates the effects of curcumin on pediatric epithelial liver tumor cells	Liver cancer	Curcumin	Blue, 480 nm	Cur-PDT enhanced anti-tumor properties by inducing loss of viability via ROS production, and inhibition of NF-κB and beta-catenin.	[41]
7	Effects of photosensitization of curcumin in human glioblastoma multiforme cells	Glioblastoma	Curcumin	Blue, 410 nm	Cur-PDT induced apoptosis through enhanced p53 expression and elevated Bax levels, which initiated the mitochondrial apoptotic pathway, promoted cytochrome c expression, and released it to inhibit the growth of glioblastoma multiforme cells.	[42]
8	Photodynamic effect of curcumin on NPC/CNE2 cells	Nasopharyngeal carcinoma	Curcumin	Blue, 400 nm	Cur-PDT induced apoptosis in NPC/CNE2 cells, either in dark cytotoxicity or photocytotoxicity, through cell membrane shrinkage.	[43]
9	Photosensitizer effect of curcumin on UVB-irradiated HaCaT cells through activation of caspase pathways	Skin cancer	Curcumin	Blue, 400 nm	Cur-PDT synergistically induced apoptosis in HaCaT cells by activating caspase-8, -3, and -9, subsequently leading to cytochrome c release.	[44]

**Table 4:** Nanotechnology of curcumin with PDT against cancer.

	Study	Types of Cancer	CHM (PS)	Types of Nano- Carrier	Light Irradiation	Consequences	Reference
1	Enhanced intracellular delivery of curcumin using polymeric nanocarriers: a natural photosensitizing agent for anti- cancer photodynamic therapy	Breast cancer	Curcumin	Polymeric	Blue, 418 nm	Cur-NPs-PDT indicated a better cytotoxic response compared to the free Cur and exhibited improved efficacy in the presence of light to inhibit the growth of breast cancer cells.	[47]
2	Effect of curcumin-nanoemulsion associated with photodynamic therapy in breast adenocarcinoma cell line	Breast adenocarcinoma	Curcumin	Nanoemulsion	Blue, 440 nm	Cur-nanoemulsion-PDT elevated caspase-3/7 activity and suppressed MCF-7 cell proliferation via ROS generation, ultimately inducing cell death through apoptotic pathways.	[48]
3	Conquering cancer multi-drug resistance using curcumin and cisplatin prodrug-encapsulated mesoporous silica nanoparticles for synergistic chemo- and photodynamic therapies	Sarcoma	Curcumin	Mesoporous silica nanoparticles	Blue, 400 nm	The Cur nanochannels of mesoporous silica nanoparticles increased the levels of ROS under light irradiation, which also acts as a structure-directing agent and p-glycoprotein inhibitor during PDT treatment to fight sarcoma cells.	[49]
4	Effect of curcumin-nanoemulsion associated with photodynamic therapy in cervical carcinoma cell lines	Cervical carcinoma	Curcumin	Nanoemulsion	Blue, 400 nm	Cur-nanoemulsion-PDT served as an alternative approach for treating cervical lesions by employing an endoscopic diode fiber laser system for either in situ or cavity activation via a diffuse fiber delivery method, resulting in elevated caspase-3/7 activity and inducing apoptosis in cervical carcinoma cells.	[50]
5	Investigation of ROS generating capacity of curcumin-loaded liposomes and its in vitro cytotoxicity on MCF-7 cell lines using photodynamic therapy	Breast cancer	Curcumin	Liposomes	Blue, 460 nm	Cur-loaded liposome enhanced ROS generation and greater cytotoxicity against cancer cells compared to free curcumin, thereby improving the PDT efficacy.	[51]
6	Treatment of breast cancer in vivo by dual photodynamic and photothermal approaches with the aid of curcumin photosensitizer and magnetic nanoparticles	Breast cancer	Curcumin	Magnetic nanoparticles	Blue 400 nm, and NIR lasers	Fe_3_O_4_/SiO_2_-Cur-PDT/PTT decreased the expression levels of apoptotic Bax and caspase 3 proteins for the treatment of triple-negative breast cancers.	[52]
7	EGFR-targeted photodynamic therapy by curcumin-encapsulated chitosan/TPP nanoparticles	Gastric cancer	Curcumin	Chitosan/TPP nanoparticles	Blue, 460 nm	Cur-encapsulated chitosan/TPP nanoparticles were a promising targeted-PDT against epidermal growth-factor-receptor-overexpressing cancers through the generation of ROS.	[53]
8	Reduction-responsive worm-like nanoparticles for synergistic cancer chemo-photodynamic therapy	Breast cancer	Curcumin	Worm-like nanoparticles	Laser, 660 nm	Cur@IR820-ss-PEG-PDT inhibited tumor angiogenesis and potentiated PDT efficacy, enhancing tumor hypoxia-inducible factor-1α and vascular endothelial cell growth factor to improve tumor accumulation and retention.	[54]
9	Two-photon photodynamic therapy with curcumin nanocomposite	Colorectal adenocarcinoma	Curcumin	Nano- composite	NIR, 900 nm	TiO_2_-Cur-PDT enhanced ROS production and penetration depth to cancer cells. Two-photon PDT was better than single-photon PDT in cell apoptosis and necrosis.	[55]
10	The comparison of in vitro photosensitizing efficacy of curcumin-loaded liposomes following photodynamic therapy on melanoma MUG-Mel2, squamous cell carcinoma SCC-25, and normal keratinocyte HaCaT cells	Skin cancer	Curcumin	Liposomes	Blue, 380–500 nm	Liposome Cur-PDT increased the ratio of apoptotic and necrotic cells, decreased malignant cell motility, and contributed to the inhibition of malignant cell metastasis.	[56]

**Table 5:** Nanogel with PDT against cancer.

	Study	Types of Cancer	PS	Types of Nanogel	Light Irradiation	Consequences	Reference
1	Facile preparation of toluidine blue-loaded DNA nanogels for anticancer photodynamic therapy	Breast cancer	Toluidine blue	DNA	LED, 660 nm	DNA/TB nanogel exhibited controlled release characteristics, efficient cellular uptake, and phototoxic effects, contributing to reduced dark toxicity in breast cancer cells.	[57]
2	Tumor-targeting nanogel that can function independently for both photodynamic and photothermal therapy and its synergy from the procedure of PDT followed by PTT	Not specific	Gold nanorods for PTT, and chlorin e6 for PDT	Chitosan- functionalized pluronic	NIR, 660 nm	A dual-function, PDT and PTT, nanosystem without quenching between PS and GNRs was successfully obtained by loading Ce6 and GNRs into the chitosan-functionalized pluronic nanogel, and it was used for the treatment of cancer.	[58]
3	pH-responsive AIE nanogels for synergistic chemo-photodynamic cancer therapy with imaging guidance	Breast cancer	BAO	PNA (poly(N-isopropylacrylamide-co-acrylic acid))	Dark or white	P@BAO-DOX nanogels offered precise drug release in acidic conditions and efficient ROS generation for imaging-guided chemo-PDT synergistic therapy for breast cancer treatment.	[59]
4	Polyphotosensitizer nanogels for GSH-responsive histone deacetylase inhibitor delivery and enhanced cancer photodynamic therapy	Prostate cancer	Chlorin e6	Histone deacetylase inhibitors	NIR, 660 nm	A nanoplatform of nanogel-Ce6 was loaded with histone deacetylase inhibitors, which enhanced synergistic therapy of prostate cancer through the inhibition of HIF-1α and vascular endothelial growth factor pathways during PDT resistance.	[60]
5	Fucoidan-based theranostic nanogel for enhancing imaging and photodynamic therapy of cancer	Fibrosarcoma	Chlorin e6	Fucoidan	NIR, 660 nm	CFN-gel exhibited a nanomolar affinity for vascular endothelial growth factor and demonstrated a significant anti-tumor effect in vivo, even without light treatment, through p-selectin targeting, which enhanced permeation and drug retention.	[61]
6	CD44-mediated tumor homing of hyaluronic acid nanogels for hypoxia-activated photodynamic therapy against tumors	Liver cancer	Chlorin e6	Hyaluronic acid	NIR, 660 nm	Hypoxia-activated hyaluronic acid nanogels generated a higher level of ROS with significant inhibition of tumor growth within the whole treatment period.	[62]
7	Peroxisome inspired hybrid enzyme nanogels for chemodynamic and photodynamic therapy	Not specific	Indocyanine green	Fe_3_O_4_ nanoparticle	NIR, 700–900 nm	Lactate oxidase and catalase in an Fe_3_O_4_ nanoparticle and indocyanine green (ICG) co-loaded hybrid nanogels significantly increased intracellular ROS levels, resulting in lethal damage to cancer cells and effectively suppressing tumor growth.	[63]
8	Bioactivatable self-quenched nanogel for targeted photodynamic therapy	Head and neck cancers	Pheophorbide A	Poly[(2-(pyridin-2-yldisulfanyl) ethyl acrylate)-co-[poly(ethylene glycol)]]	Laser, 670 nm	A bioactivatable self-quenching nanogel overexpressed epidermal growth factor receptor in the tumor with better tumor targeting efficiency and PDT effect.	[64]
9	Real-time monitoring of colorectal cancer location and lymph node metastasis and photodynamic therapy using fucoidan-based therapeutic nanogel and near-infrared fluorescence diagnostic therapy system	Colorectal cancer	Chlorin e6 and 5-aminolevulinic acid	Fucoidan	NIR, 660 nm	CFN-gel showed a high accumulation efficiency in cancer cells and high fluorescence signals in near-infrared light, and only CFN-gel slowed tumor progression by reducing its size during the treatment of PDT.	[65]
10	Modulation of glutathione levels by redox-active nanogel carriers for the synergistic enhancement of photodynamic therapy	Not specific	Chlorin e6	Fe_3_O_4_ nanoclusters	NIR, 660 nm	The composite nanogel demonstrated enhanced cellular uptake, prolonged circulation time, and targeted tissue distribution, efficient intracellular degradation, and selective cytotoxicity, leading to ROS generation that promoted apoptosis and inhibited tumor growth.	[66]

**Table 6:** Preparation of a nanogel with curcumin against cancer.

	Study	Types of Cancer	CHM (PS)	Types of Nanogel	Consequences	Reference
1	Curcumin-encapsulating nanogels as an effective anticancer formulation for intracellular uptake	Breast cancer	Curcumin	Amphiphilic poloxamer-cationic	Nanogel carriers offered an innovative way to encapsulate curcumin, which was a more effective anticancer therapeutic for specific tumor targeting, such as using antibodies against the surface receptors specific to breast cancer cells.	[67]
2	Anti-breast cancer activity of pH-responsive nanogel loaded with curcumin	Breast cancer	Curcumin	Chitosan-carboxymethyl-β-cyclodextrin	Cur-loaded chitosan-CM-β-CD nanogel effectively inhibited the growth of breast cancer cells, as it depended on the pH value.	[68]
3	Curcumin loaded chitin nanogels for skin cancer treatment via the transdermal route	Skin cancer	Curcumin	Chitin	The curcumin-loaded chitin nanogels demonstrated a 4-fold enhancement in the steady transdermal flux of curcumin, accompanied by loosening of the stratum corneum, which facilitated deeper skin penetration without eliciting any signs of inflammation—supporting its potential for melanoma treatment.	[69]
4	Anticancer-drug-based multifunctional nanogels through self-assembly of dextran–curcumin conjugates toward cancer theranostics	Cervical cancer	Curcumin	Dextran	The dextran-curcumin nanoparticles were effectively delivered into HeLa cells and exhibited anticancer activity and strong fluorescence available for live-cell imaging.	[70]
5	pH-responsive magnetic CuFe_2_O_4_-PMAA nanogel conjugated with amino-modified lignin for controlled breast cancer drug delivery	Breast cancer	Curcumin	CuFe_2_O_4_@ poly(methacrylic acid)	CuFe_2_O_4_@PMAA@Lig-ADH@Cur exhibited significant cytotoxic effects. Its release profile was pH-dependent, exhibiting an accelerated release rate under acidic conditions, thereby contributing to the inhibition of breast cancer.	[71]
6	Enhanced anticancer response of curcumin- and piperine-loaded lignin-g-p (NIPAM-co-DMAEMA) gold nanogels against U-251 MG glioblastoma multiforme	Brain cancer	Curcumin, piperine	Lignin-g-p (NIPAM-co-DMAEMA) gold	Curcumin- and piperine-loaded lignin-g-p (NIPAM-co-DMAEMA) gold nanogels showed better internalization or association with the cancer cells, and penetrated the cells via endocytic pathways, as well as induced the apoptosis of the related caspase-3.	[72]
7	An improved method in fabrication of smart dual-responsive nanogels for controlled release of doxorubicin and curcumin in HT-29 colon cancer cells	Colon cancer	Curcumin	Doxorubicin	The doxorubicin and curcumin nanogels induced cell apoptosis in HT-29 colon cancer cells, representing superior antitumor efficacy compared to single-drug formulations or free drugs, attributed to the controlled release properties.	[73]
8	Fmoc-FF nanogel-mediated delivery of doxorubicin and curcumin in thyroid cancer cells	Thyroid cancer	Curcumin	Doxorubicin	Nα-9-fluorenylmethoxycarbonyl-diphenylalanine peptide-based nanogels loaded with doxorubicin and curcumin were internalized into thyroid cancer cell lines and predominantly localized within the cytoplasm instead of early endosomes, thereby maintaining intracellular stability.	[74]
9	Self-assembled thermoresponsive nanogel from grafted hyaluronic acid as a biocompatible delivery platform for curcumin with enhanced drug loading and biological activities	Breast, liver and skin cancers	Curcumin	Hyaluronic acid-grafted poly(N-isopropylacrylamide) (HA-pNIPAM)	The curcumin-loaded HA-pNIPAM nanogel exhibited anti-proliferative effects against cancer cells, which enhanced the aqueous solubility and inhibited the TNF-α pathway.	[75]
10	Curcumin-loaded Arabic gum aldehyde-gelatin nanogels for breast cancer therapy	Breast cancer	Curcumin	Aldehyde-gelatin	The curcumin-loaded nanogels induced selective toxicity in MCF-7 cells because high encapsulation efficiency improved their bioavailability and therapeutic effectiveness against cancer cells.	[76]

**Table 7:** Curcumin-loaded nanogel with PDT against cancer or disease.

	Study	Types of Diseases	CHM (PS)	Types of Nanogel	Light Irradiation	Consequences	Reference
1	Enhanced nanogel formulation combining natural photosensitizer curcumin and pectis brevipedunculata (*Asteraceae*) essential oil for synergistic daylight photodynamic therapy in leishmaniasis treatment	Neglected tropical diseases	Curcumin	F127/Carbopol 974P	Blue, 460 nm	The nanogel formulation nGPC incorporating EOPb/Cur represented a promising therapeutic approach against *Leishmania* (LLa) promastigotes under dynamic PDT conditions, effectively addressing the limitations of conventional leishmaniasis treatments by enhancing drug stability, minimizing toxicity, and enabling controlled release of bioactive compounds for targeted action.	[77]
2	Stimuli-responsive, plasmonic nanogel for dual delivery of curcumin and photothermal therapy for cancer treatment	Breast cancer	Curcumin	AuNP@/Cur	NIR Laser, 808 nm	Plasmonic nanogel loaded with curcumin acted as a stimuli-responsive nanocarrier, having potential for dual therapy, such as delivery of a hydrophobic drug and photothermal therapy to inhibit the breast cancer cells.	[78]

## Data Availability

No new data were created or analyzed in this study. Data sharing is not applicable to this article.
